# Sugar-sweetened beverage consumption and attention-deficit/hyperactivity disorder symptoms in children and adolescents: a systematic review and meta-analysis of observational studies

**DOI:** 10.3389/fnut.2026.1858738

**Published:** 2026-09-16

**Authors:** Bin Shi, Ming-jun Ma, Miao Yang, Yu-qiang Li

**Affiliations:** 1 College of Physical Education and Health, East China Normal University, Shanghai, China; 2 Key Laboratory of Adolescent Health Assessment and Exercise Intervention of the Ministry of Education, East China Normal University, Shanghai, China; 3 The First Clinical Medical College, Anhui University of Chinese Medicine, Hefei, Anhui, China

**Keywords:** ADHD, children and adolescents, hyperactivity, neurodevelopmental disorders, sugar-sweetened beverages

## Abstract

**Background:**

Attention-deficit/hyperactivity disorder (ADHD) is a common neurodevelopmental disorder in children and adolescents. Previous studies have suggested a potential association between sugar-sweetened beverage (SSB) consumption and ADHD or related symptoms; however, existing evidence remains inconsistent, and the magnitude of the association and sources of heterogeneity have not been comprehensively evaluated. Therefore, we conducted a systematic review and meta-analysis to examine the association between SSB intake and ADHD or related symptoms in children and adolescents.

**Methods:**

We systematically searched PubMed, Embase, Web of Science, APA PsycINFO, and the Cochrane Library from inception to January 2026 for observational studies assessing the association between SSB consumption and ADHD or related symptoms. Adjusted effect estimates, including odds ratios (ORs), hazard ratios (HRs), or risk ratios (RRs), with corresponding 95% confidence intervals (CIs) were extracted and converted to ORs as the common summary measure. Random-effects meta-analyses were performed, with 95% CIs calculated using the Hartung-Knapp adjustment. Between-study heterogeneity was assessed using the I^2^ statistic. Potential publication bias or small-study effects were evaluated using Doi plots and the Luis Furuya-Kanamori (LFK) index. Prespecified subgroup analyses and sensitivity analyses were conducted to explore sources of heterogeneity and assess the robustness of the findings.

**Results:**

Nine observational studies involving 461,109 children and adolescents were included. The meta-analysis showed that higher SSB consumption was significantly associated with an increased likelihood of ADHD or related symptoms (OR = 1.23, 95% CI: 1.02–1.48). Moderate heterogeneity was observed across studies (I^2^ = 63.3%, τ^2^ = 0.0434, *p* = 0.0053), and the prediction interval crossed the null value (0.79–1.90), indicating uncertainty in effect estimates for future studies. Subgroup analyses demonstrated largely consistent effect directions across most strata but did not fully explain the observed heterogeneity. Leave-one-out sensitivity analyses showed that the direction of the association remained positive, although exclusion of Kim et al., attenuated the pooled estimate (OR = 1.16, 95% CI: 1.00–1.34) and reduced heterogeneity (I^2^ = 42.1%), indicating some influence of this study on the magnitude and heterogeneity of the pooled association. Doi plot analysis revealed marked asymmetry, with an LFK index of 3.37, indicating possible small-study effects or publication bias.

**Conclusion:**

This systematic review and meta-analysis suggests that higher consumption of sugar-sweetened beverages is associated with an increased likelihood of ADHD or related symptoms in children and adolescents. However, because all included evidence was derived from observational studies and was characterized by moderate heterogeneity, a prediction interval crossing the null, and potential publication bias, the findings should be interpreted with caution. Further high-quality prospective studies are needed to clarify the independent association and underlying mechanisms linking SSB intake to ADHD-related symptoms.

**Systematic review registration:**

https://www.crd.york.ac.uk/PROSPERO/view/CRD420251186577, identifier CRD420251186577.

## 1 Introduction

Attention-deficit/hyperactivity disorder (ADHD) is a common neurodevelopmental disorder with onset in childhood, characterized by persistent patterns of inattention, hyperactivity, and impulsivity that are inconsistent with developmental level. ADHD not only impairs academic performance, emotional and behavioral regulation, peer relationships, and family functioning in children and adolescents, but also persists into adolescence and adulthood in a substantial proportion of individuals, resulting in long-term individual and public health burdens (1, 2). Recent systematic reviews estimate a pooled prevalence of approximately 7.6% among children aged 3–12 years and 5.6% among adolescents aged 12–18 years. However, considerable heterogeneity exists across diagnostic criteria, regions, and study designs, suggesting that beyond genetic susceptibility, modifiable environmental factors may contribute to the epidemiology of ADHD (2, 3).

Among the various potentially modifiable environmental exposures, sugar-sweetened beverages (SSBs) have attracted sustained attention because of their widespread prevalence, relatively clear definition, and clear public health actionability. The World Health Organization (WHO) defines free sugars as monosaccharides and disaccharides added to foods by manufacturers, cooks, or consumers, as well as sugars naturally present in honey, syrups, fruit juices, and fruit juice concentrates. It recommends that both children and adults limit free sugar intake to less than 10% of total energy intake, with a further reduction to below 5% conferring additional health benefits (4, 5). Nevertheless, a global analysis covering 185 countries reported a 22.9% increase in mean SSB consumption among children and adolescents aged 3–19 years between 1990 and 2018. In 2018, 56 of 185 countries had a mean SSB intake of ≥7 servings per week among children and adolescents aged 3–19 years, representing approximately 238 million young people globally (6).

From a mechanistic perspective, the association between SSB consumption and ADHD-related phenotypes is biologically plausible. Compared with total carbohydrate intake, recent systematic reviews suggest that carbohydrate quality–particularly exposure to added sugars, SSBs, refined sugars, and high glycemic load–is more consistently associated with ADHD-related symptom burden (7). Previous studies have proposed that high sugar intake, especially fructose loading, may alter impulsivity and attentional control by influencing rewards-related dopaminergic signaling, energy metabolism, and inflammation-related pathways (8). Moreover, ADHD is closely linked to sleep disturbances and circadian rhythm disruptions (2, 9), suggesting that high-sugar dietary exposures may influence ADHD-related symptoms through interacting neurobiological and behavioral pathways (8, 9). Although these mechanisms require further empirical validation, they provide testable theoretical support for epidemiological observations (7–9). Epidemiological evidence has gradually accumulated, but findings remain inconsistent. A case-control study from Taiwan reported a dose-response relationship between SSB consumption and physician-diagnosed ADHD in children, indicating approximately a fourfold higher likelihood of ADHD among children consuming ≥7 servings per week (10). A Norwegian mother-child cohort study found that maternal intake of sugar-sweetened carbonated beverages during pregnancy was associated with an increase in ADHD symptoms in offspring (11). A nationwide administrative cohort study in South Korea further showed that higher SSB consumption before the age of 2 years was associated with an elevated risk of subsequent ADHD (12). A cross-sectional study among school-aged children in China also observed dose-response associations between SSB intake frequency and hyperactive behaviors (13). In contrast, a Brazilian birth cohort study did not identify a clear association between changes in sucrose intake from 6 to 11 years of age and incident ADHD (14). Consistent with these findings, a 2020 systematic review and meta-analysis reported a positive association between overall “sugar or sugar-sweetened beverage” exposure and ADHD symptoms. However, only 7 observational studies were included, heterogeneity was substantial, and subgroup analyses suggested that “dietary sugar alone” was not stably associated with ADHD. These findings imply that SSBs, as a specific exposure category, may have epidemiological significance distinct from total sugar intake (15). In addition, meta-analyses of broader dietary patterns have shown that “unhealthy dietary patterns” characterized by high intakes of refined sugars and saturated fats are associated with a higher risk of ADHD, suggesting that SSBs may act both as an independent exposure and as a marker of overall poor dietary quality (16).

Therefore, a focused systematic review and meta-analysis examining the association between SSB consumption and ADHD symptoms in children and adolescents is warranted. Compared with pooling diverse forms of sugar exposure, isolating SSB intake offers three key advantages. First, SSBs represent a relatively clearly defined exposure that can be directly translated into public health policies and dietary recommendations. Second, several new cohort and cross-sectional studies have been published since 2020, warranting an updated synthesis of the evidence. Third, existing studies differ substantially in exposure assessment, outcome definition, developmental time windows, and confounder adjustment, underscoring the need for systematic integration to evaluate the overall strength and robustness of the association (6, 10–16). Accordingly, this study aims to systematically evaluate the association between SSB consumption and ADHD symptoms among children and adolescents in observational studies, and to quantitatively synthesize the existing evidence through meta-analysis, thereby providing more robust evidence to inform dietary interventions and public health prevention strategies.

## 2 Materials and methods

This systematic review was reported in accordance with the Preferred Reporting Items for Systematic Reviews and Meta-Analyses (PRISMA) 2020 statement and supplemented by the Meta-analysis of Observational Studies in Epidemiology (MOOSE) checklist to address items specific to observational studies. In addition, this review was prospectively registered in the International Prospective Register of Systematic Reviews (PROSPERO) under the registration number CRD420251186577. No amendments were made to the registered protocol after registration.

### 2.1 Search strategy

We systematically searched PubMed, Embase, Web of Science Core Collection, APA PsycINFO, and the Cochrane Library for studies reporting associations between attention-deficit/hyperactivity disorder (ADHD) and sugar-sweetened beverages (SSBs). The search covered each database from inception to January 2026, with no language restrictions.

To enhance search sensitivity, terms related to artificially sweetened beverages (ASBs) were also included; however, studies in which ASBs were the primary exposure were excluded during the screening stage. In addition, reference lists of all included articles were manually screened to identify further relevant studies.

### 2.2 Eligibility criteria

Inclusion criteria were as follows:

(1) Participants were children or adolescents aged ≤18 years;

(2) The study explicitly reported associations between SSB consumption and ADHD or ADHD-related symptoms;

(3) The study design was observational, including cross-sectional, cohort, or case-control studies;

(4) Adjusted effect estimates [odds ratios (ORs), hazard ratios (HRs), or risk ratios (RRs)] with corresponding 95% confidence intervals (CIs) were available or could be derived.

Exclusion criteria were:

(1) Conference abstracts, commentaries, reviews, dissertations, animal studies, and interventional studies;

(2) Studies that did not report adjusted effect estimates or lacked sufficient data for quantitative synthesis;

(3) Studies conducted exclusively in adult populations or those reporting only maternal SSB intake during pregnancy;

(4) Studies with unclear exposure or outcome definitions that could not be included in quantitative meta-analysis.

### 2.3 Study selection and data extraction

Study selection and data extraction were independently performed by two investigators. Duplicate records were first removed using EndNote 20. Title/abstract screening and full-text eligibility assessment were conducted manually within EndNote 20 by the two investigators. No machine-learning prioritization, re-ranking algorithms, or AI-assisted screening tools were used during the study selection process. Discrepancies were resolved through discussion; if consensus could not be reached, a third investigator made the final decision. Data extraction was conducted using Microsoft Excel and included study characteristics (first author, publication year, country/region, and study design), sample size and age range, exposure assessment methods, outcome measurement approaches, and reported effect estimates with corresponding 95% confidence intervals.

Most included studies reported adjusted ORs, whereas only one cohort study reported an adjusted HR. Given the low baseline event risk in this cohort (approximately 2%), the HR was treated as an approximation of the RR and subsequently converted to the OR scale using the RR–OR relationship described by Zhang and Yu (17). All pooled analyses were therefore conducted using adjusted ORs.

For studies reporting only stratified results (e.g., by sex) rather than an overall effect estimate, and given that these stratified estimates are derived from the same study population, we combined them into a single study-level effect estimate using inverse-variance weighting prior to the final analysis. This approach, in accordance with established meta-analytic principles, avoids double-counting and preserves the independence of effect sizes (18).

### 2.4 Quality assessment

Given the inclusion of different types of observational studies, methodological quality was assessed using design-specific tools. For cohort and case-control studies, the Newcastle-Ottawa Scale (NOS) was applied (19). This scale evaluates study quality across three domains: selection of study groups, comparability between groups, and outcome assessment, with a maximum score of 9 points; studies scoring 7–9 points were considered high quality.

For cross-sectional studies, quality was assessed using the criteria recommended by the Agency for Healthcare Research and Quality (AHRQ) (20). This scale consists of 11 items, each scored as 1 (“yes”) or 0 (“no” or “unclear”). Based on commonly used thresholds, total scores of 8–11 indicated high quality, 4–7 moderate quality, and 0–3 low quality.

All quality assessments were conducted independently by two investigators. Disagreements were resolved through discussion, with arbitration by a third investigator when necessary.

### 2.5 Statistical analysis

All statistical analyses were performed using R software (version 4.5.0) (21), primarily employing the meta and metasens packages (22). Considering potential heterogeneity across studies in terms of design, population characteristics, and exposure and outcome assessment methods, random-effects models were applied to pool effect estimates. Between-study variance (τ^2^) was estimated using the Paule-Mandel method (23), and 95% CIs were adjusted using the Hartung-Knapp approach to improve robustness, particularly under conditions of a limited number of studies (24).

Statistical heterogeneity was assessed using Cochran’s Q test (*p* < 0.10) and quantified using the I^2^ statistic, with I^2^ values of 0%, 25%, 50%, and 75% generally representing no, low, moderate, and high heterogeneity, respectively (25). The τ^2^ statistic was also reported to reflect the magnitude of between-study variability in true effects.

To explore potential sources of heterogeneity and examine the influence of study characteristics, prespecified subgroup analyses were conducted according to outcome assessment method (ADHD-specific assessment/diagnosis vs. hyperactivity symptom scales), exposure assessment method (parent-reported vs. self-reported or mixed), beverage type (soft drinks vs. SSBs), age group (≤6 years, >6 to ≤12 years, >12 years), and sex (boys vs. girls).

Sensitivity analyses were performed using a leave-one-out approach to evaluate the robustness of pooled estimates (26). Given the relatively small number of included studies, conventional funnel plots and Egger’s test were considered unreliable; therefore, publication bias was assessed using Doi plots and the Luis Furuya-Kanamori (LFK) index (27). Due to substantial variability in exposure definitions, intake units, and exposure categorization across studies, a dose-response meta-analysis was not conducted (28). Forest plots were used to display study-specific and pooled effect estimates, while subgroup results were summarized in tabular form with corresponding forest plots provided in the Supplementary material.

## 3 Results

### 3.1 Literature search and study characteristics

A total of 360 records were identified through the systematic search, including 44 from PubMed, 75 from Web of Science, 163 from Embase, 66 from APA PsycINFO, and 12 from the Cochrane Library. After removing 101 duplicate records, 259 articles were screened based on titles and abstracts, of which 197 were excluded for not meeting the eligibility criteria. Full-text assessment was subsequently conducted for 62 articles. During full-text screening, 53 articles were excluded for the following reasons: review articles (*n* = 26), conference abstracts (*n* = 2), meta-analyses (*n* = 1), editorials or commentaries (*n* = 1), dissertations (*n* = 1), animal studies (*n* = 5), adult populations (*n* = 8), maternal SSB intake during pregnancy rather than SSB intake in children or adolescents [*n* = 1; Kvalvik et al. (11)], failure to report extractable effect estimates (*n* = 4), and provision of crude estimates only (*n* = 4). Ultimately, nine studies were included in the quantitative synthesis (meta-analysis) (Figure 1).

**FIGURE 1:**
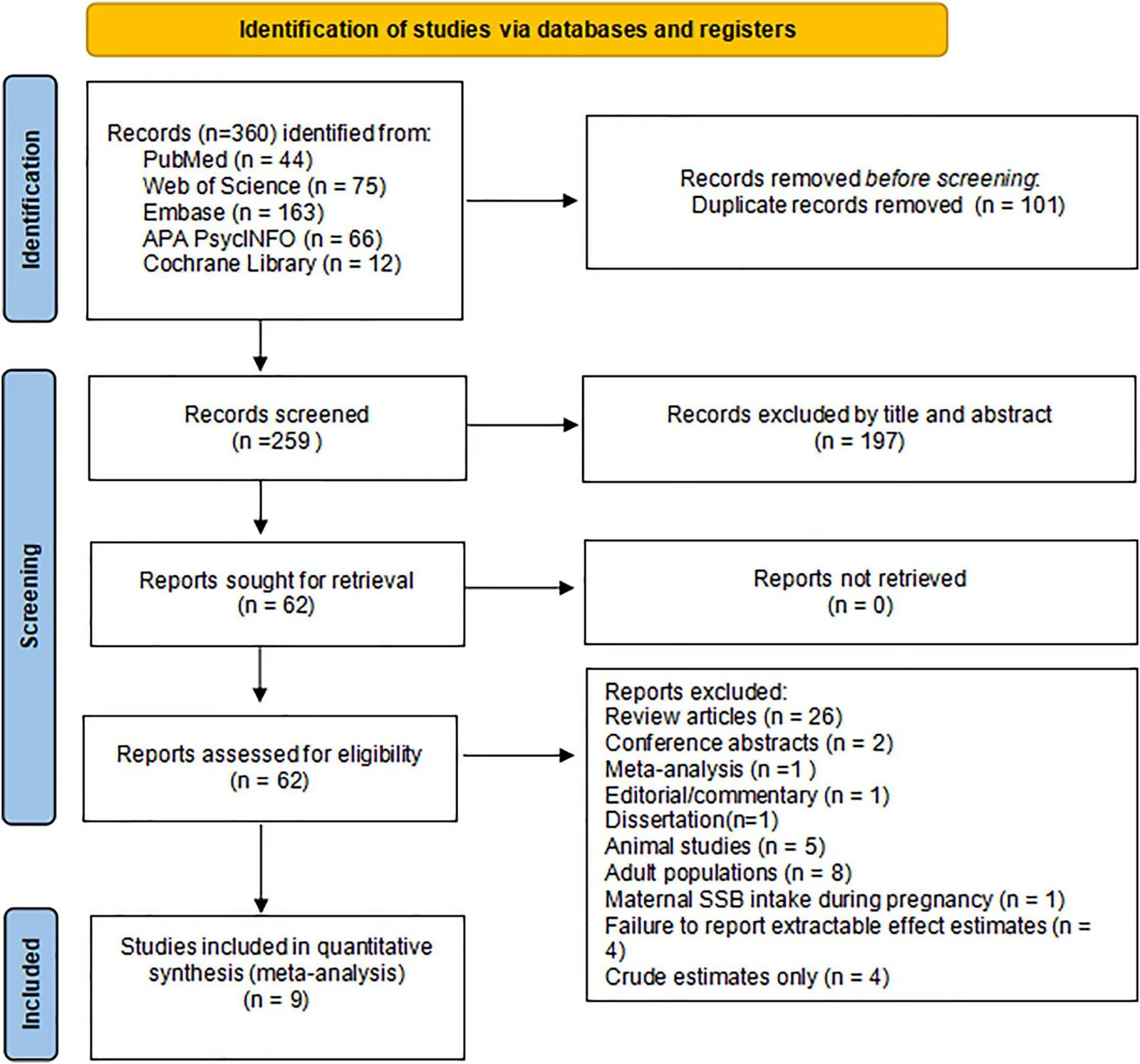
Flow chart of the search and selection process.

This systematic review included nine observational studies, comprising seven cross-sectional studies, one case-control study, and one cohort study. The included studies were published between 2006 and 2024 and were conducted in mainland China (13, 29–31), Taiwan Province of China (10), South Korea (12, 32), Norway (33), and the United States (34). The age of participants ranged from 1.5 to 16 years, with a total sample size of 461,109 individuals. All studies included both male and female participants.

Regarding exposure definition, some variability was observed across studies. Seven studies assessed SSBs as an aggregate exposure (10, 12, 13, 29–31, 34), whereas two studies specifically evaluated soft drink consumption (32, 33). Exposure definitions and intake thresholds varied across studies, including daily or weekly intake, comparisons between high- and low-frequency consumption, and analyses using continuous exposure variables.

With respect to outcome assessment, a range of instruments was used to evaluate ADHD or related symptoms in children and adolescents, including the Strengths and Difficulties Questionnaire (SDQ) (29–31, 33, 34), Korean ADHD Rating Scale (K-ARS) (32), International Classification of Diseases (ICD) diagnostic codes (F90.0, F90.1, F90.8, and F90.9) (12), DSM-IV-TR diagnostic criteria (10), and the Conners Teacher Rating Scale-Revised: Short Form (CTRS-R:S) (13). Six studies used hyperactivity symptom scales or related behavioral symptom measures as outcomes (13, 29–31, 33, 34), whereas three studies used ADHD-specific assessments or diagnostic criteria as the primary outcome (10, 12, 32).

Regarding exposure assessment, all included studies assessed SSB consumption using questionnaire-based methods, including reports from parents or caregivers, self-reports from children and adolescents, health screening questionnaires, simplified food frequency questionnaires (FFQs), and 24-h dietary recalls. Notably, the included studies varied considerably in terms of the reporter, questionnaire format, and the units or frequency metrics used to quantify SSB intake.

In terms of effect estimate reporting, most studies provided overall effect estimates, while some additionally reported sex-stratified results; a few studies reported effect estimates for only one sex. For the primary meta-analysis, overall effect estimates were preferentially extracted where available. When only stratified estimates were reported, available subgroup-specific estimates were combined as described in the Methods. Specific exposure assessment methods and study characteristics are presented in Table 1.

**TABLE 1:** Characteristics of the included studies reporting the association between SSBs and ADHD.

Study/ year	Country	Design	Age range/ mean age	Sample size	Exposure	Consumed amount (Exposure vs. Reference)	Exposure assessment	Adjusted OR (95% CI)	Outcome/ assessment tool	Adjustments	Quality assessment score
Geng et al. (29)	China	Cross-sectional	3–6/4.5	27,200	SSB	≥2 times/day vs. <1 time/day	a	1.11 (0.99–1.26)	Hyperactivity/SDQ	1, 2, 5–6, 8–10, 12, 18–19	8/11
Kim et al. (32)	Korea	Cross-sectional	6–12/9.29	16,831	SD	≥1 time/day vs. never	a	1.75 (1.33–2.29)	ADHD/K-ARS	2, 5–6, 11	8/11
Kim et al. (12)	Korea	Cohort	18–24 months /1.75	365,236	SSB	≥200 mL/day vs. <200 mL/day	a	1.17 (1.08–1.28)	ADHD/ ICD-10 code	5, 11–12, 21, 23, 27–28	9/9
Lien et al. (33)	Norway	Cross-sectional	15–16 /15.5	5,498	SD	>4 glasses/day vs. 1–6 glasses/week	b	Boys: 1.99 (1.23–3.24) Girls: 0.99 (0.47–2.10)	Hyperactivity/SDQ	6–7, 13–15, 17, 20, 24	7/11
Yu et al. (10)	China, Taiwan	Case-control	4–15/cases: 9.2 controls: 8.9	cases: 173 controls: 159	SSB	≥7 times/week vs. 0 times/week	a	3.69 (1.29–10.60)	ADHD/ DSM-IV-TR	1, 6, 14, 22, 25–26	9/9
Schwartz et al. (34)	USA	Cross-sectional	6–18/12.4	1,649	SSB	Continuous (per 1 drink/day increase)	b	1.14 (1.06–1.22)	Hyperactivity/SDQ	2–3, 5, 7, 11, 16	9/11
Zhang et al. (13)	China	Cross-sectional	6–12/8.6	6,541	SSB	≥2 times/week vs. 0 times/week	a	1.07 (0.82–1.39)	Hyperactivity/ CTRS-R:S	2, 5, 7, 10–14, 17	9/11
Zhang et al. (30)	China	Cross-sectional	6–18/12.4	30,188	SSB	High frequency vs. low frequency	Both a and b	1.03 (0.93–1.14)	Hyperactivity/SDQ	1, 4–6, 28–29	8/11
Zhou et al. (31)	China	Cross-sectional	3–6/4.69	7,634	SSB	≥2 times/day vs. <1 time/day	a	1.15 (1.00–1.32)	Hyperactivity/SDQ	2, 5–6, 8–12	7/11

Exposure: SSB, sugar-sweetened beverages; SD, soft drinks. Exposure assessment: (a) questionnaires/reports completed by caregivers or parents; (b) online health surveys/self-reports completed by participants. Adjustments: 1. Gender; 2. Age; 3. Race/ethnicity; 4. Grade; 5. Family economic situation (school lunch eligibility); 6. Parental education level; 7. Family structure/single-child status; 8. Screen time; 9. Sleep duration; 10. Physical activity time/outdoor activity; 11. Sex; 12. Body mass index (BMI); 13. Smoking status/parental smoking status; 14. Consumption of milk/meat/fruit/vegetables/dairy foods/fish or fish products; 15. Regular consumption of lunch and breakfast; 16. Sugary food consumption; 17. Consumption of potato chips/chocolates/sweets/deep-fried food; 18. Plain water consumption; 19. Parents’ parenting behaviors (supportive/engaged and hostile/coercive); 20. Perceived social support; 21. Birth weight; 22. Maternal alcohol consumption during pregnancy; 23. Early infancy feeding type; 24. History of intoxication; 25. Family history of nervous system diseases; 26. DRD4 gene polymorphism at rs752306; 27. Birth calendar year; 28. Birth region/residential area; 29. Academic record.

### 3.2 Main meta-analysis results

Nine observational studies were included in the primary meta-analysis. Using a random-effects model with Hartung-Knapp adjustment, higher SSB consumption was significantly associated with an increased likelihood of ADHD or related symptoms among children and adolescents (pooled OR = 1.23, 95% CI: 1.02–1.48) (Figure 2). Moderate heterogeneity was observed across studies (I^2^ = 63.3%, τ^2^ = 0.0434, *p* = 0.005), and the prediction interval ranged from 0.79 to 1.90, indicating uncertainty in the magnitude of the association in future studies. Therefore, subgroup and sensitivity analyses were conducted to explore potential sources of heterogeneity and to assess the robustness of the findings.

**FIGURE 2:**
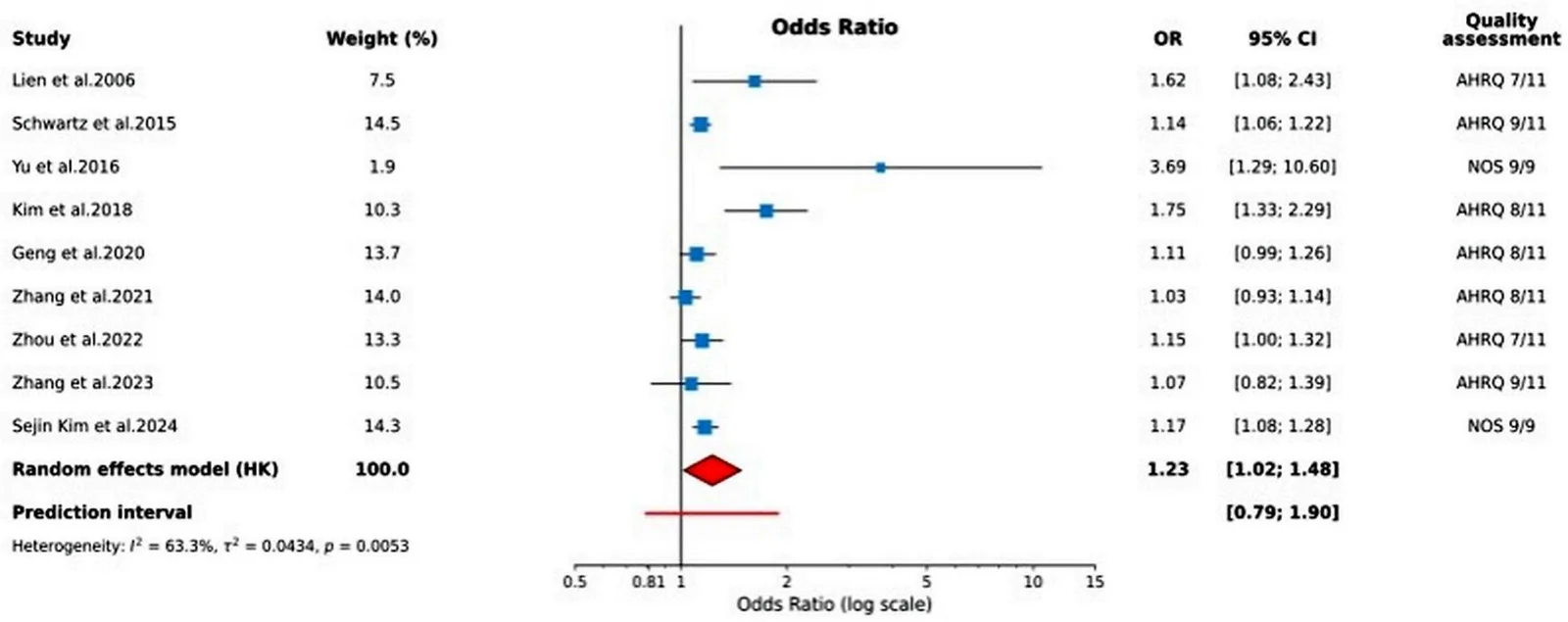
Forest plot of the association between sugar-sweetened beverage consumption and ADHD or related symptoms in children and adolescents. Methodological quality scores are shown for each study using the Agency for Healthcare Research and Quality (AHRQ) criteria for cross-sectional studies and the Newcastle–Ottawa Scale (NOS) for cohort and case-control studies.

### 3.3 Subgroup analysis results

To investigate potential sources of heterogeneity, prespecified subgroup analyses were performed according to outcome assessment method, exposure assessment method, beverage type, age group, and sex. Overall, the direction of the pooled effect estimates was generally consistent across most subgroups, suggesting a positive association between higher SSB intake and ADHD or related symptoms in children and adolescents. However, several subgroup estimates should be interpreted cautiously because of the small number of studies, wide confidence intervals, and substantial residual heterogeneity.

Table 2 summarizes the pooled ORs, 95% CIs, and heterogeneity statistics for each subgroup analysis, with the corresponding forest plots presented in Supplementary Figures 1–5.

**TABLE 2:** Subgroup analyses of the association between SSB consumption and ADHD or related symptoms.

Subgroup	Studies (n)	Pooled OR (95% CI)	I^2^ (%)	P for heterogeneity within subgroup
Overall	9	1.23 (1.02–1.48)	63.3	0.005
Outcome assessment				
Hyperactivity symptom scale	6	1.11 (1.03–1.21)	20.2	0.281
ADHD-specific assessment/diagnosis	3	1.67 (0.49–5.72)	83.3	0.0025
Exposure assessment				
Self-reported / mixed	3	1.17 (0.72–1.90)	66.5	0.051
Parent-reported	6	1.28 (0.93–1.77)	64.9	0.014
Beverage type				
Soft drinks	2	1.71 (1.09–2.69)	0.0	0.757
Sugar-sweetened beverages	7	1.12 (1.02–1.24)	33.4	0.173
Age group				
≤6 years	3	1.15 (1.07–1.23)	0.0	0.783
>6 to ≤12 years	3	1.66 (0.42–6.53)	79.7	0.007
>12 years	3	1.17 (0.72–1.90)	66.5	0.051
Sex				
Boys	4	1.42 (0.70–2.88)	65.3	0.035
Girls	3	1.14 (0.94–1.39)	0.0	0.658

Results for subgroups with few studies and wide confidence intervals after Hartung-Knapp adjustment should be interpreted with caution.

#### 3.3.1 Outcome assessment subgroup

When stratified by outcome assessment method, studies using hyperactivity symptom scales showed a significant positive association between higher SSB intake and ADHD-related symptoms (OR = 1.11, 95% CI: 1.03–1.21), with low heterogeneity (I^2^ = 20.2%, τ^2^ = 0.0016, *p* = 0.281). In contrast, studies employing ADHD-specific assessments or diagnostic criteria yielded a pooled OR of 1.67 (95% CI: 0.49–5.72). Although the direction of association was positive, statistical significance was not reached, and heterogeneity was substantial (I^2^ = 83.3%, τ^2^ = 0.1874, *p* = 0.0025; Supplementary Figure 1). Overall, while effect directions were broadly consistent, the limited number of studies, wide confidence intervals, and high heterogeneity in the ADHD-specific subgroup mean that the current evidence remains insufficient to establish a clear modifying effect of outcome assessment method on this association.

#### 3.3.2 Exposure assessment subgroup

When stratified by exposure assessment method, the pooled OR for studies using self-reported or mixed reporting was 1.17 (95% CI: 0.72–1.90), indicating a positive but non-significant association, with moderate-to-high heterogeneity (I^2^ = 66.5%, τ^2^ = 0.029, *p* = 0.051). For studies using parent-reported exposure assessment, the pooled OR was 1.28 (95% CI: 0.93–1.77), also non-significant and accompanied by high heterogeneity (I^2^ = 64.9%, τ^2^ = 0.074, *p* = 0.014; Supplementary Figure 2). These findings do not provide clear evidence that exposure assessment method materially modifies the association between SSB intake and ADHD symptoms.

#### 3.3.3 Beverage type subgroup

In subgroup analyses by beverage type, higher soft drink consumption was associated with an increased risk of ADHD symptoms (OR = 1.71, 95% CI: 1.09–2.69), with no observed heterogeneity (I^2^ = 0.0%, τ^2^ = 0.000, *p* = 0.757). Similarly, the SSB subgroup demonstrated a positive association (OR = 1.12, 95% CI: 1.02–1.24), with low-to-moderate heterogeneity (I^2^ = 33.4%, τ^2^ = 0.007, *p* = 0.173; Supplementary Figure 3). Although the effect estimate was larger in the soft drink subgroup, this subgroup included only two studies, and the findings should therefore be interpreted cautiously.

#### 3.3.4 Age group subgroup

When stratified by age, higher SSB intake was significantly associated with greater odds of ADHD-related symptoms among children aged ≤6 years (OR = 1.15, 95% CI: 1.07–1.23), with no evident heterogeneity (I^2^ = 0.0%, τ^2^ = 0.000, *p* = 0.783). Among children aged >6 to ≤12 years, the pooled effect estimate was higher but imprecise and non-significant (OR = 1.66, 95% CI: 0.42–6.53), with high heterogeneity (I^2^ = 79.7%, τ^2^ = 0.233, *p* = 0.007). In adolescents aged >12 years, the association remained positive but was not statistically significant (OR = 1.17, 95% CI: 0.72–1.90), with moderate-to-high heterogeneity (I^2^ = 66.5%, τ^2^ = 0.029, *p* = 0.051; Supplementary Figure 4). Although the ≤6-year subgroup showed the most stable association, current evidence is insufficient to draw definitive conclusions regarding age-dependent effects.

#### 3.3.5 Sex subgroup

In sex-stratified analyses, the pooled OR among boys was 1.42 (95% CI: 0.70–2.88), indicating a positive but non-significant association with moderate-to-high heterogeneity (I^2^ = 65.3%, τ^2^ = 0.141, *p* = 0.035). Among girls, the pooled OR was 1.14 (95% CI: 0.94–1.39), also non-significant, with no observed heterogeneity (I^2^ = 0.0%, τ^2^ = 0.000, *p* = 0.658; Supplementary Figure 5).

Overall, available evidence does not support a clear sex-specific modification of the association between SSB consumption and ADHD symptoms.

### 3.4 Sensitivity analysis

Sensitivity analyses using a leave-one-out approach showed that the pooled effect estimates remained consistently positive, with ORs ranging from 1.16 to 1.27, indicating that the main findings were not driven by any single study. Exclusion of the study by Kim et al. (32) resulted in the lowest pooled estimate (OR = 1.16, 95% CI: 1.00–1.34) and reduced heterogeneity (I^2^ = 42.1%), suggesting that this study had some influence on both the overall effect size and between-study heterogeneity.

In several leave-one-out analyses, the lower bound of the 95% CI approached or slightly fell below unity, reflecting the conservative nature of the Hartung-Knapp adjustment. Overall, the results demonstrate a reasonable degree of robustness but should be interpreted cautiously in light of the limited number of studies and residual heterogeneity (Figure 3).

**FIGURE 3:**
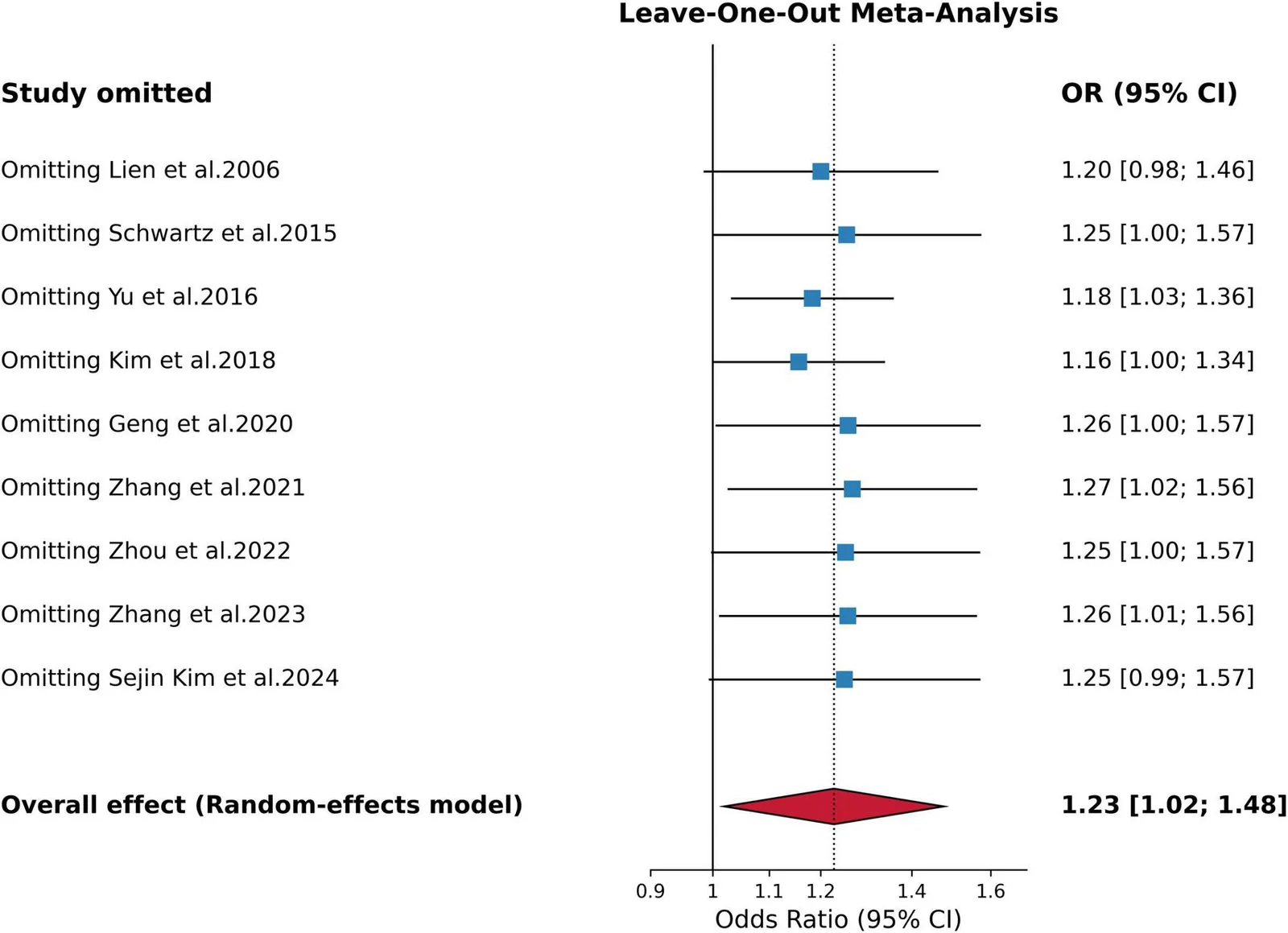
Leave-one-out sensitivity analysis of the pooled association between sugar-sweetened beverage consumption and ADHD or related symptoms.

Additional influence diagnostics are presented in Supplementary Figure 6 (Baujat plots) and Supplementary Figure 7 (cumulative meta-analysis plots). Baujat plots further indicated that Kim et al. (32) contributed substantially to both overall heterogeneity and the pooled effect estimate, while Yu et al. (10), Zhang et al. (30), and Lien et al. (33) also exerted moderate influence. Therefore, we further address potential sources of heterogeneity in the Discussion by integrating study design, exposure definitions, and outcome assessment across included studies (Supplementary Figure 6).

### 3.5 Assessment of publication bias

Potential publication bias or small-study effects were evaluated using Doi plots and the Luis Furuya-Kanamori (LFK) index. The Doi plot demonstrated marked asymmetry, with an LFK index of 3.37, indicating major asymmetry and suggesting the presence of potential small-study effects or publication bias (Figure 4). However, given the small number of included studies, these findings should be interpreted with caution.

**FIGURE 4:**
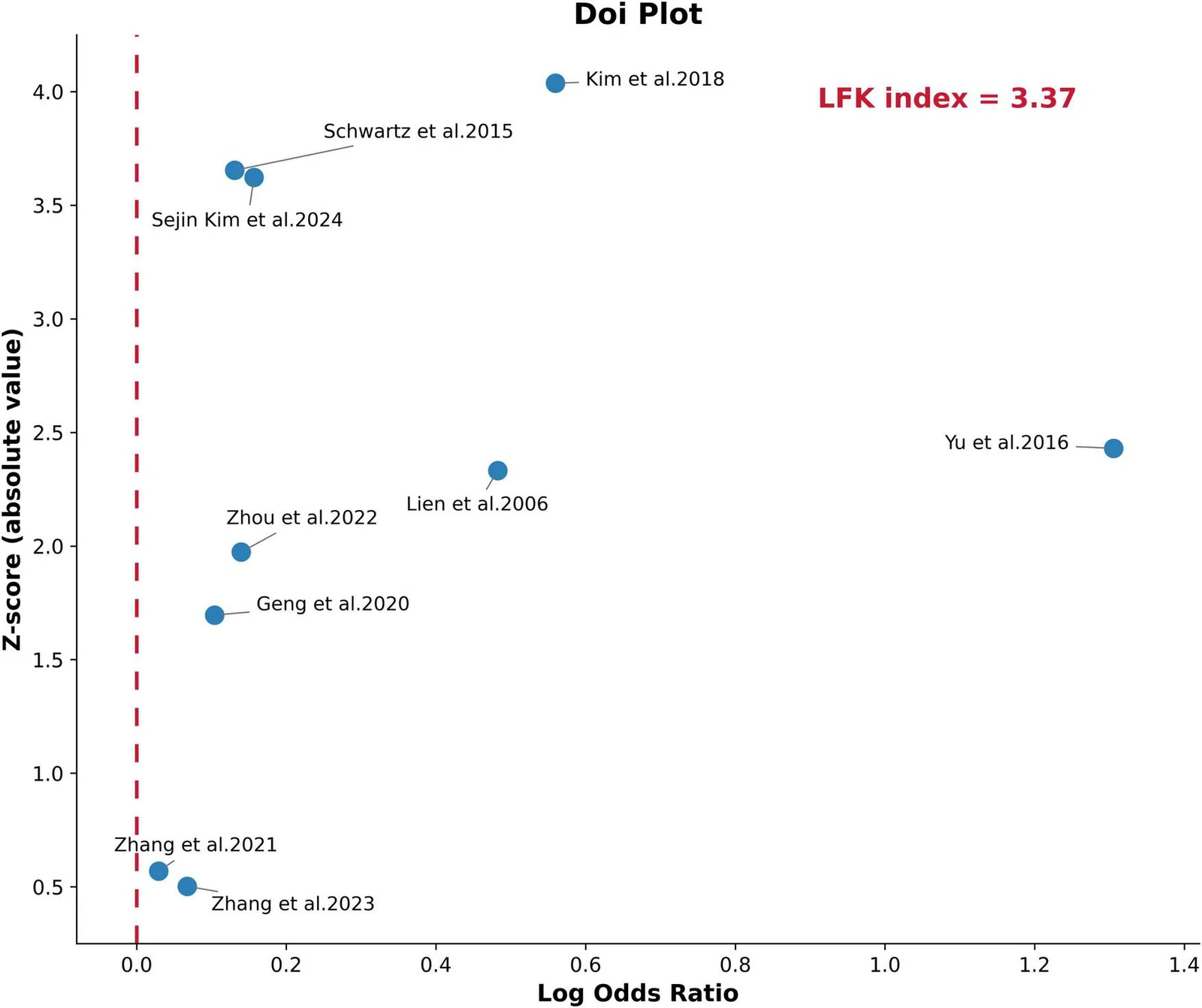
Doi plot and LFK index assessing potential publication bias or small-study effects.

## 4 Discussion

This systematic review and meta-analysis synthesized the available observational evidence on the association between SSB consumption and attention-deficit/hyperactivity disorder (ADHD) or related hyperactivity symptoms in children and adolescents. The pooled analysis showed that higher levels of SSB intake were significantly associated with an increased likelihood of ADHD or related symptoms (OR = 1.23). Sensitivity analyses further indicated that the direction of this association was generally robust. Given that childhood and adolescence represent critical periods for neurodevelopment and the formation of behavioral patterns, these findings suggest that daily dietary exposures, particularly higher SSB consumption, may be relevant to neurobehavioral health in children and adolescents and therefore warrant further attention.

Overall, the findings of this study are consistent with previous systematic reviews examining the relationships between sugar intake, overall dietary patterns, and ADHD symptoms. Prior studies have suggested that higher sugar consumption, unhealthy dietary patterns, and junk food intake may all be associated with an elevated risk of ADHD in children and adolescents (15, 16, 35). Unlike earlier studies that primarily focused on total sugar intake, ultra-processed foods, or overall dietary patterns, the present study specifically targeted SSBs as a more clearly defined exposure with direct public health relevance. As SSBs constitute a major source of free sugar intake among children and adolescents and are relatively easy to identify and modify, these findings may provide more targeted evidence to inform future nutritional intervention strategies. Nevertheless, the present results should not be interpreted as evidence of a definitive causal relationship between SSB consumption and ADHD, but rather as indicating an epidemiological association that merits further investigation.

In recent years, a growing number of studies have suggested that ADHD should not be viewed solely as a neuropsychiatric disorder, but rather as a complex systemic condition arising from the combined effects of genetic factors, nutritional exposures, metabolic regulation, and environmental factors (36).

Within this broader disease framework, long-term high SSB consumption may act as an adverse nutritional exposure that influences neurodevelopment and behavioral performance in children through multiple biological pathways. First, SSB intake has been proposed to induce rapid elevations in blood glucose and exaggerated insulin responses, followed by glycemic fluctuations, which may impair prefrontal executive function and attentional control (37, 38). Second, high sugar exposure may enhance immediate rewards sensitivity within the nucleus accumbens-prefrontal rewards circuitry and exacerbate abnormalities in rewards prediction error processing, thereby promoting impulsive behavioral tendencies (39, 40). In addition, chronic high sugar intake may trigger low-grade inflammation and oxidative stress via the gut-brain axis, subsequently affecting synaptic plasticity and neurodevelopmental processes (41, 42).

However, the observed association may not solely reflect the direct effects of sugar intake itself. Growing evidence indicates that SSB consumption in children often serves as a marker of broader dietary patterns and food environment characteristics. A recent study covering more than 150 countries reported that healthier national-level food supply structures were associated with lower ADHD prevalence, suggesting that the overall dietary environment may exert a systemic influence on ADHD risk (43). Similarly, another review emphasized that the development and progression of ADHD may be closely linked to overall dietary patterns, family eating behaviors, and broader social environmental changes, rather than being determined by a single nutritional factor (44).

At the same time, the possibility of reverse causality warrants careful consideration. Neuropsychological studies have shown that children with ADHD tend to exhibit higher delay discounting and stronger preferences for immediate rewards (40). Animal model studies have further demonstrated that impulsive decision-making behaviors observed in ADHD may be related to functional abnormalities in the medial prefrontal cortex-nucleus accumbens circuitry. Reduced functional coupling within this circuit may predispose individuals to preferentially select high-sugar, energy-dense foods to obtain rapid rewards stimulation (45). Cross-sectional studies have also demonstrated significant associations between ADHD symptoms and the risk of disordered eating, with impulsivity and inattention potentially influencing eating patterns (46). Other studies have reported that individuals with ADHD consume sugar-sweetened beverages, processed foods, and snacks more frequently than healthy controls, and that these unhealthy dietary patterns are significantly associated with ADHD symptom severity (47). Associations between dietary patterns and ADHD symptoms, as well as comorbid behaviors in early childhood, further suggest that overall dietary structure may exert a systemic influence on ADHD risk (48). Given that the majority of studies included in this review employed cross-sectional designs, with exposure and outcome assessed at the same time point, the current evidence does not allow for clear determination of causal direction. Therefore, the observed associations should be interpreted cautiously as statistical associations rather than causal relationships.

A moderate level of heterogeneity was observed in this meta-analysis (I^2^ = 63.3%), indicating moderate variability among the included studies. Subgroup analyses did not fully account for the sources of heterogeneity. Influence diagnostics and leave-one-out sensitivity analyses further suggested that certain individual studies exerted a disproportionate impact on the overall heterogeneity and pooled effect estimate. Among them, the study by Kim et al. (32) had the most pronounced influence. Exclusion of this study resulted in a substantial reduction in heterogeneity, suggesting that it may represent one of the primary contributors to between-study variability. This study included a relatively large sample of Korean primary school children aged 6–12 years, assessed ADHD symptoms using the parent-reported K-ARS, and treated soft drinks as one component of an unhealthy dietary pattern. Its comparatively higher effect estimate, together with its greater statistical weight, likely amplified its influence on the overall pooled result.

In addition, the studies by Yu et al. (10), Zhang et al. (30), and Lien et al. (33) may also have influenced the overall effect estimate to varying degrees. Yu et al. (10) employed a case-control design and defined ADHD based on physician diagnosis according to DSM-IV-TR criteria, resulting in a relatively strong contrast between exposure groups and effect estimates that were notably higher than those reported in most other studies. In contrast, the effect estimate reported by Zhang et al. (30) was close to the null value and may have exerted a downward influence on the pooled effect. This study used the SDQ to assess broader psychological and behavioral problems, and its outcome definition was therefore not fully aligned with ADHD-specific diagnoses or symptom scales. The study by Lien et al. (33), conducted in Norway among adolescents aged 15–16 years, defined exposure as sugar-containing soft drinks, with high intake categorized as more than 4 servings per day. Its regional context, age distribution, exposure threshold, and outcome assessment differed substantially from those of most studies conducted among younger Asian populations. Taken together, the observed heterogeneity in this meta-analysis is likely attributable to differences in study design, regional background, participant age, exposure definition and intake thresholds, outcome specificity and measurement instruments, and strategies for confounder adjustment. In particular, the use of ADHD-specific diagnostic criteria in some studies and broader hyperactivity or behavioral symptom measures in others may have contributed to between-study variability. Therefore, although the direction of the pooled association was generally consistent across studies, the overall findings should be interpreted with caution in light of these multiple sources of heterogeneity.

With respect to publication bias, the Doi plot in combination with the LFK index indicated marked asymmetry (LFK = 3.37), suggesting the potential presence of small-study effects or underlying bias. However, in meta-analyses of observational studies, graphical asymmetry does not necessarily equate to true publication bias (27). Substantial between-study differences in study design, exposure measurement precision, outcome assessment criteria, and confounder control strategies may also contribute to asymmetrical distributions of effect estimates. Given that the number of included studies remains relatively limited, the application of bias-adjustment methods at this stage may itself yield unstable estimates. Therefore, although the possibility of an overestimation of the pooled effect cannot be entirely excluded, the current evidence is insufficient to conclude that the observed association is primarily driven by publication bias.

From a broader public health perspective, the findings of this study have some practical implications. A large body of studies has consistently demonstrated that SSB consumption is closely associated with increased risks of childhood obesity, type 2 diabetes, metabolic syndrome, and cardiovascular disease (49). The WHO has therefore explicitly recommended that children should limit their intake of free sugars as much as possible (4). The present study further suggests that, beyond metabolic health, SSB consumption may also be linked to neurobehavioral health in children and adolescents. Importantly, these findings should not be interpreted in a simplistic manner as evidence that “sugar causes ADHD.” A more plausible interpretation is that frequent SSB consumption serves as a marker of broader dietary and environmental patterns, including increased ultra-processed food intake, imbalanced dietary structure, and exposure to unhealthy food environments. These factors may jointly influence long-term health and developmental trajectories in children.

This study has several notable strengths. First, it specifically focused on sugar-sweetened beverage intake as a relatively well-defined exposure with clear public health relevance, rather than broadly examining total sugar intake or overall dietary patterns. This focus enhances the specificity and translational value of the findings. Second, a total of nine observational studies were included, comprising more than 460,000 children and adolescents, which improved the statistical power of the pooled effect estimates. Third, given the limited number of eligible studies, random-effects models combined with Hartung-Knapp adjustments were applied to calculate confidence intervals, resulting in more conservative and robust effect estimates. Fourth, the robustness of the findings, potential sources of heterogeneity, and the presence of publication bias were comprehensively evaluated using multiple complementary approaches, including subgroup analyses, leave-one-out sensitivity analyses, cumulative meta-analysis, Baujat plots, and Doi plots. Together, these analyses provide a more comprehensive interpretation of the results.

Several limitations of this study should be acknowledged. First, all included studies were observational in nature, with most adopting a cross-sectional design. Observational studies were included because the review aimed to synthesize epidemiological evidence on the association between habitual SSB consumption and ADHD-related outcomes in real-world settings. As a result, causal relationships between SSB intake and ADHD or related symptoms cannot be established, and the possibility of reverse causation cannot be excluded. Second, substantial variability existed across studies with respect to study design, participant age, regional background, exposure definitions, intake thresholds, reference categories, and outcome measurement tools, which may have contributed to between-study heterogeneity. To avoid unduly narrowing the available evidence base, eligibility was not restricted to a single observational study design or to uniform exposure and outcome assessment methods. This broader eligibility approach allowed a more comprehensive synthesis of the available evidence but reduced comparability across studies and likely contributed to the observed heterogeneity. For example, some studies defined outcomes based on clinical ADHD diagnoses, whereas others assessed hyperactivity or broader psychological and behavioral problems, leading to inconsistencies in outcome definitions. Third, the definitions and classifications of SSBs varied across studies, with some evaluating SSBs and others focusing on soft drinks. In addition, intake frequency and quantity were categorized using inconsistent thresholds, which precluded the conduct of a formal dose-response meta-analysis. Fourth, exposure assessment relied largely on self-reports by children or adolescents or proxy reports by parents, introducing the potential for recall bias and exposure misclassification. Fifth, the extent of confounder adjustment differed substantially between studies. Key factors such as physical activity, sleep duration, screen time, body mass index, total energy intake, family socioeconomic status, and overall diet quality were not consistently or adequately controlled for in all studies, raising the possibility of residual confounding. Sixth, only nine studies were included in the present meta-analysis, and the number of studies within certain subgroups was small. This limited the statistical power of subgroup analyses and constrained the ability to fully explain heterogeneity. Overall, although the included studies were generally rated as moderate to high methodological quality according to the design-specific assessment tools, the predominance of cross-sectional designs and inconsistencies in confounder adjustment remain important limitations of the evidence base. Moreover, although the Doi plot and LFK index suggested the potential presence of small-study effects or publication bias, this finding should be interpreted cautiously given the limited number of included studies. Therefore, the overall findings of this study should be interpreted with appropriate caution in light of these limitations.

## 5 Conclusion

This systematic review and meta-analysis suggests that higher intake of sugar-sweetened beverages is associated with an increased likelihood of ADHD or related symptoms in children and adolescents. However, given that the current evidence is predominantly derived from observational studies and is characterized by moderate heterogeneity, potential reverse causation, residual confounding, and possible publication bias, these findings should be interpreted with caution. Future research should prioritize high-quality prospective cohort studies employing standardized exposure assessment, outcome measurement, and confounder control strategies to further clarify the independent association and underlying mechanisms linking SSB consumption to ADHD-related symptoms in children and adolescents.

## Data Availability

The raw data supporting the conclusions of this article will be made available by the authors, without undue reservation.
