# Supplementary material for: Sugar-sweetened beverage consumption and attention-deficit/hyperactivity disorder symptoms in children and adolescents: a systematic review and meta-analysis of observational studies
Source: Front Nutr. 2026 Sep 16;13:1858738. doi: 10.3389/fnut.2026.1858738 (PMC13623580; doi:10.3389/fnut.2026.1858738)
Supplement: Supplementary file 1 [file Table_1.docx]

**Supplementary Figure 1. Outcome assessment subgroup**

**Supplementary Figure 2. Exposure assessment subgroup**

**Supplementary Figure 3. Beverage type subgroup**

**Supplementary Figure 4. Age group subgroup**

**Supplementary Figure 5. Sex subgroup**

**Supplementary Figure 6. Baujat Plot**

**Supplementary Figure 7. Cumulative Meta-Analysis**
